# A pre-catalytic non-covalent step governs DNA polymerase β fidelity

**DOI:** 10.1093/nar/gkz1076

**Published:** 2019-11-16

**Authors:** Khadijeh S Alnajjar, Ivan S Krylov, Amirsoheil Negahbani, Pouya Haratipour, Boris A Kashemirov, Ji Huang, Mariam Mahmoud, Charles E McKenna, Myron F Goodman, Joann B Sweasy

**Affiliations:** 1 Department of Cellular and Molecular Medicine, University of Arizona, Tucson, AZ 85724, USA; 2 Department of Chemistry, University of Southern California, Los Angeles, CA 90089, USA; 3 Department of Biological Sciences, University of Southern California, Los Angeles, CA 90089, USA; 4 University of Arizona Cancer Center, Tucson, AZ 85724, USA

## Abstract

DNA polymerase β (pol β) selects the correct deoxyribonucleoside triphosphate for incorporation into the DNA polymer. Mistakes made by pol β lead to mutations, some of which occur within specific sequence contexts to generate mutation hotspots. The adenomatous polyposis coli (APC) gene is mutated within specific sequence contexts in colorectal carcinomas but the underlying mechanism is not fully understood. In previous work, we demonstrated that a somatic colon cancer variant of pol β, K289M, misincorporates deoxynucleotides at significantly increased frequencies over wild-type pol β within a mutation hotspot that is present several times within the APC gene. Kinetic studies provide evidence that the rate-determining step of pol β catalysis is phosphodiester bond formation and suggest that substrate selection is governed at this step. Remarkably, we show that, unlike WT, a pre-catalytic step in the K289M pol β kinetic pathway becomes slower than phosphodiester bond formation with the APC DNA sequence but not with a different DNA substrate. Based on our studies, we propose that pre-catalytic conformational changes are of critical importance for DNA polymerase fidelity within specific DNA sequence contexts.

## INTRODUCTION

DNA polymerase β (pol β) functions during base excision repair (BER) to fill small DNA gaps that are created during the removal of damaged bases (1–3). Pol β is also necessary to generate genomic diversity during VDJ recombination and has been recently shown to fill gaps in the DNA during alternative non-homologous end-joining (4,5). The BER pathway is responsible for the removal of 20 000–50 000 lesions per cell per day (6), meaning that mistakes committed during BER can lead to cancer-driving mutations. Therefore, it is imperative that pol β selects the correct dNTP from a pool of similarly structured dNTPs during the gap-filling step of BER, given its central role in this repair pathway.

To provide mechanistic insights into substrate selection by pol β, we first characterized catalytically active mutator variants of this enzyme that we identified in genetic screens and in tumors (for examples see (7–12)). The majority of these pol β mutations do not map to its active site. This suggests that the fidelity of pol β is likely to be governed by amino acid residues that are distant from the active site of the enzyme. In addition, our findings indicate that the pol β mutator variants exhibit sequence context-specific misincorporation of nucleotides at hotpots that differ from wild-type pol β (WT pol β) (13–18).

The mechanism of substrate selectivity by DNA pol β has been the subject of numerous studies. It was previously suggested that substrate selectivity by pol β occurs during phosphodiester bond formation, which is the rate-determining step of the reaction (19–24). This is based on the observed ‘thio effect’ by DNA pol β, utilizing a dNTP analogue modified by substituting a nonbridging oxygen with sulfur (25,26). The difference in the magnitude of the ‘thio effect’ in the presence of either the correct or incorrect nucleotide indicated that phosphodiester bond formation is rate-determining. Moreover, studies utilizing dNTP analogues modified at the β, γ-bridging group have shown that phosphodiester bond formation is rate-determining and is, therefore, important for selectivity (19,22,23). Other studies, including our own, have provided evidence that in addition to phosphodiester bond formation, pre-catalytic conformational changes facilitate an induced fit mechanism of selectivity by pol β (for examples see (27–30)). Pre-catalytic conformational changes, albeit fast, have been shown in kinetic detail to be important for selectivity (31–33).

**Figure 1.**
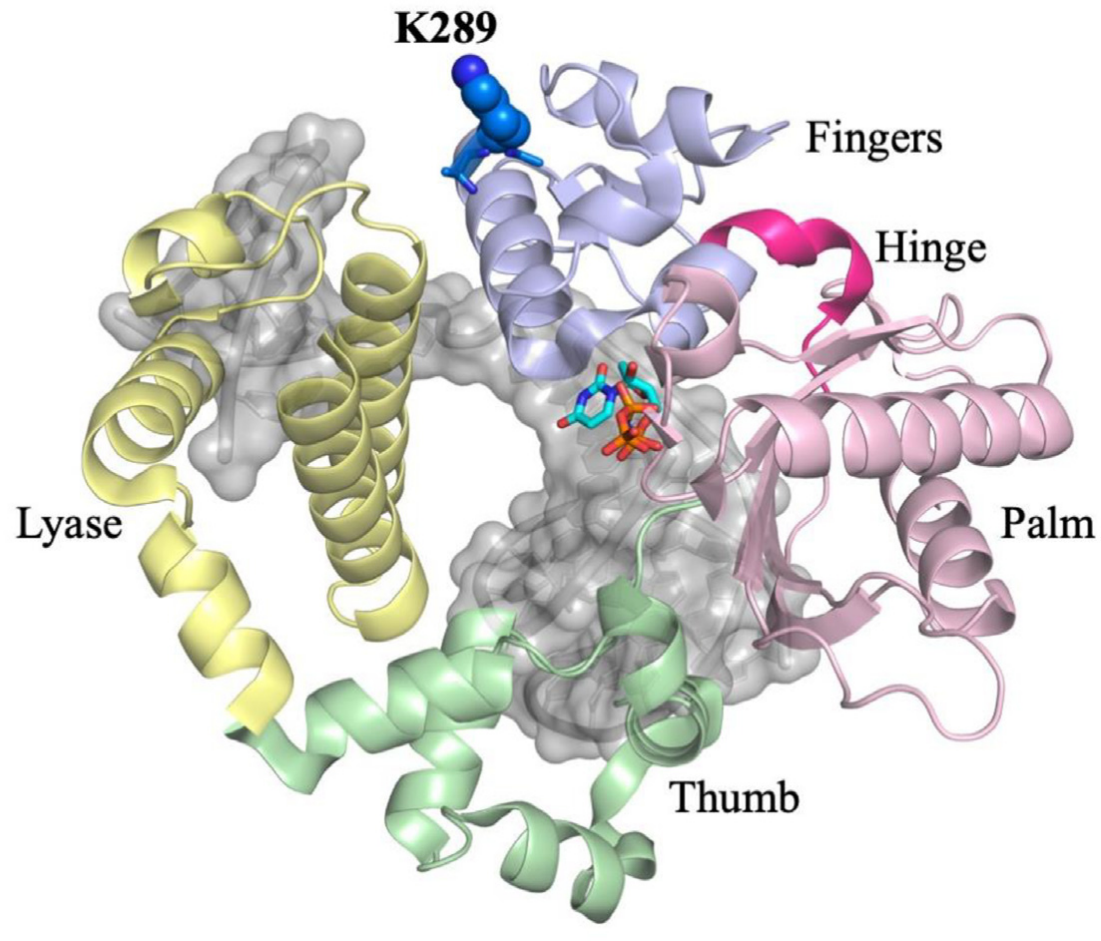
The three-dimensional structure of the closed ternary complex of DNA polymerase β. The structure is organized into catalytic domains, in yellow is the lyase N-terminal domain. The polymerase domain contains three subdomains, the thumb (green), palm (pink), and fingers (light blue) subdomains. K289 (blue) is located on the distal end of helix N of the fingers subdomain, away from the active site.

This work focuses on the K289M pol β variant, which was identified in colorectal tumors and has been shown to induce mutations within specific sequence contexts (34). Lysine 289 resides at the end of helix N of pol β, which is part of the fingers subdomain (Figure 1). We previously demonstrated that K289M induces mutations specifically within the AACAA sequence context that is present at 22 sites within the adenomatous polyposis coli (APC) gene, and which is mutated in 80% of familial colorectal cancers (35). A recent study from our laboratory suggested that, unlike WT pol β, the rate-determining step of the reaction is not dependent on the chemical property of the incoming dNTP in the context of the AACAA DNA sequence (16). Here, we provide evidence that phosphodiester bond formation is rate-limiting for K289M with a control sequence, but that it may not be rate-limiting for the AACAA DNA sequence. Therefore, a pre-catalytic conformational step within the reaction pathway could be slower than the rate of phosphodiester bond formation during DNA synthesis when K289M utilizes a DNA substrate containing the AACAA sequence context. Interestingly, the K289M pol β variant exhibits a similar misincorporation specificity as the WT enzyme. Thus, substrate selectivity is likely governed by the underlying mechanism(s) of correct incorporation by pol β K289M (16). Here, we probe the sequence-context dependence of the reaction mechanism of K289M using Förster resonance energy transfer (FRET) and a subset of the ‘nucleotide analogue toolkit’ (19,22). We provide evidence that a pre-catalytic non-covalent step (NCS) is slower than phosphodiester bond formation for K289M during DNA synthesis with the AACAA template but not with a control primer-template.

## MATERIALS AND METHODS

### Protein expression and purification

WT and K289M pol β were expressed and purified as previously described (16).

### DNA substrates and dNTP analogues

Oligonucleotides were purchased from the Keck Oligo Synthesis Resource at Yale University and were purified by polyacrylamide gel electrophoresis. The sequences used in this study are reported in Table 1 and were prepared as previously described (16). The dGTP analogues were synthesized as previously reported (19,22,36). This subset of analogues was selected because it contains analogues that can probe the transition state (TS) and that closely represent the structure of the parent nucleotide, binding the enzyme with a similar affinity as the parent nucleotide.

**Table 1.** DNA substrates

Name	Sequence
Control	5′ GCCTATTACCGCGCAGATGCGC GTCGGAACAACGCATGCCGTCC
	CGGATAATGGCGCGTCTACGCGCCAGCCTTGTTGCGTACGGCAGG 5′
APC	5′ GCCTCGAACTCCATATGGATTT TTCAGAACGCTCGGTTGCGTCC
	CGGAGCTTGAGGTATACCTAAACAAGTCTTGCGAGCCAACGCAGG 5′
Control in APC	5′ GCCTCGAACTCCATATGGATGC GTCAGAACGCTCGGTTGCGTCC
	CGGAGCTTGAGGTATACCTACGCCAGTCTTGCGAGCCAACGCAGG 5′
APC in control	5′ GCCTATTACCGCGCAGATGCTT TTCGGAACAACGCATGCCGTCC
	CGGATAATGGCGCGTCTACGAACAAGCCTTGTTGCGTACGGCAGG 5′

The bold T is the position of the Dabcyl dT. The five nucleotides in bold near the gap (red and blue) were tested for sequence specificity. The underlined C is the templating base.

### Single turnover polymerase activity

A solution containing 50 nM DNA and 200 nM polymerase was rapidly mixed with varying concentrations of dNTP at 37 °C in 50 mM Tris pH 8.0, 20 mM NaCl, 10 mM MgCl_2_, 2 mM DTT and 10% glycerol using a KinTek rapid-quench flow apparatus. Reactions were quenched with EDTA and the product was separated from the initial reactant on a 20% polyacrylamide gel containing 8 M urea. Rate of phosphodiester bond formation (*k*_pol_) and the apparent dNTP dissociation constant (*K*_D_) were extracted as previously reported (16). Fidelity was calculated using the catalytic specificity (*k*_pol_/*K*_D_) for correct (dGTP) and incorrect (dCTP): (*k*_pol_/*K*_D_)_correct_/(*k*_pol_/*K*_D_) _incorrect_ (37).

### Förster resonance energy transfer (FRET)

**Scheme 1.**
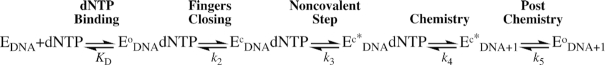
Reaction mechanism of DNA polymerization by DNA polymerase β.

Pol β containing C239S, C267S, V303C, was labeled with the fluorophore IAEDANS at position V303C as has been previously reported (27). DNA substrate containing a Dabcyl labeled dT at the −8 position relative to the templating base was used to quench the AEDANS emission. AEDANS-labeled Pol β (400 nM) and the Dabcyl-labeled DNA substrate (200 nM) were mixed with varying dGTP concentrations at 37°C using an Applied Photophysics stopped-flow fluorometer as previously reported (27). The reverse reaction was performed by rapidly mixing pre-formed ternary complex (400 nM AEDANS-labeled pol β, 200 nM dideoxy terminated Dabcyl-labeled DNA, 1 μM dGTP, 10 mM MgCl_2_) with 10-fold excess of non-labeled binary complex containing extendable DNA using a stopped-flow fluorometer. The fluorescence signal was detected over a period of 50 s. Each reaction was repeated twice with at least 10 shots for each repeat. This assay was performed in order to observe fingers re-opening upon extraction of the dGTP by the dark binary complex. Since the DNA used in the trap is extendable, we assumed that this reaction is irreversible. Results from the competition assay were fitted to a two exponential rate equation using GraphPad Prism. All the results were modeled using KinTek Global Explorer (41) in accordance to Scheme 1 (15,27).

FitSpace Explorer was used to ensure that the values of our model were constrained by the data (38). Our model follows a sequential reaction mechanism where dNTP binds to the binary complex of pol β (Scheme 1) and induces a large subdomain motion, termed fingers closing, followed by the NCS. These steps have been suggested to lead to optimum active site geometry, which is important for efficient phosphodiester bond formation (27). The *K*_D(dNTP)_ values were constrained with the biochemically measured values (Supplementary Table S1), additionally, the reverse values of fingers closing and NCS were constrained with values from the competition assay (Supplementary Table S2). We also assumed that pyrophosphorolysis is negligible and is therefore constrained to zero. The values for the forward conformational changes (fingers closing and NCS) along with the rates of phosphodiester bond formation and post-chemistry steps were estimated from the fits. Confidence contour analysis was performed in order to ensure that the model parameters were constrained by the data.

## RESULTS

We utilized different primer-template sequences (Table 1) in order to provide molecular insights into the sequence context-dependent mutagenesis of the K289M pol β cancer-associated variant. All sequences contain C as the templating base. The control sequence (Table 1) is traditionally used in structural studies of pol β (28,39). The APC sequence (Table 1) is a well-known mutational hotspot in APC-mutated colorectal cancer and is also a hotspot for mutation during DNA synthesis by K289M pol β (34,35). The fidelity of K289M is only ∼2-fold lower than WT pol β with the control sequence but 6.4-fold lower with the APC sequence (Table 2, Supplementary Table S1. The *K*_D(DNA)_ for WT pol β is 2–3 nM for each of these sequences whereas it is 3–5 nM for K289M (Supplementary Figure S1, Supplementary Table S3).

**Table 2.** Sequence specificity of WT and K289M fidelity

		Template: incoming	Fidelity (×10^4^)^a^	Loss in fidelity relative to WT control^b^
WT	Control	C:C	12	1.0
	APC	C:C	2.4	4.8
	Control in APC	C:C	20	0.6
	APC in Control	C:C	7.9	1.5
	AAGAA	G:G	8.7	1.3
	AAAAA	A:G	2.0	5.6
K289M	Control	C:C	5.9	1.9
	APC	C:C	0.4	31.1
	Control in APC	C:C	5.3	2.2
	APC in Control	C:C	1.5	7.5
	AAGAA	G:G	7.9	1.5
	AAAAA	A:G	2.0	5.7

^a^Fidelity is calculated from (*k*_pol_/*K*_D_)_correct_/(*k*_pol_/*K*_D_)_incorrect_.

^b^WT Fidelity with control/fidelity value.

### K289M exhibits sequence context-specificity at the transition state

**Figure 2.**
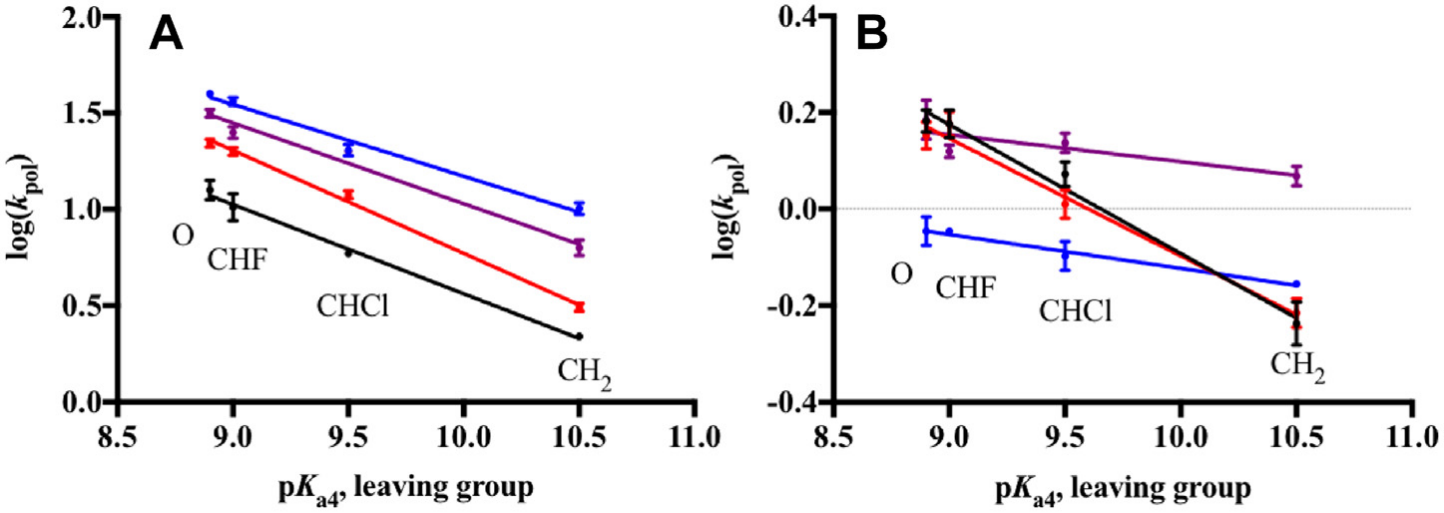
Sequence context-specificity of the linear free energy relationship for correct dGTP incorporation. The relationship between log(*k*_pol_) and p*K*_a4_ of the leaving group of different dGTP analogues for WT (**A**) and K289M (**B**) is shown for the Control (black), APC (blue), Control in APC (red) and APC in Control (purple) sequences. Data points were fit to a linear equation using GraphPad Prism. WT and K289M have the following LFER slopes, respectively: Control (−0.46, −0.27), APC (−0.41, −0.07), Control in APC (−0.52, −0.24), and APC in Control (−0.42, −0.06).

DNA pol β catalyzes the formation of a highly charged chemical TS, which forms when the 3′ hydroxyl of the primer DNA attacks the α phosphate of the incoming dNTP (40). Altering the basicity of the pyrophosphate leaving group (p*K*_a4_) by modifying the β, γ-bridging group of the incoming dNTP enables us to probe the effect of leaving group charge stability on the chemical TS (19,22,23). Because log(*k*_pol_) reflects the free energy of the chemical TS, its correlation with the p*K*_a4_ of the leaving group in a linear free energy relationship (LFER) can be profitably examined. In this model, the slope of the LFER reflects the sensitivity of the TS to variation in charge stability of the leaving group, with insensitivity shown by a zero slope and sensitivity by a negative slope. Based on this criterion and other evidence (20,21), DNA pol β was shown to catalyze a chemical TS that is destabilized by more basic leaving groups during both correct and incorrect incorporation, though to different extents, consistent with a rate-limiting phosphodiester bond formation step (16,19,22,23). As shown in Figure 2, the chemical TS of WT pol β is insensitive to the sequence context during the correct incorporation of dGTP analogues opposite template C for the control and APC sequences (Supplementary Table S4), as shown by LFER slopes of −0.46 (Figure 2A, black) and −0.41 (Figure 2A, blue), respectively.

The LFER slope of K289M exhibits sequence context-specificity. In the presence of the control sequence, K289M has a negative LFER slope (−0.27, Figure 2B, black), suggesting that leaving group basicity has an effect on the K289M rate-limiting TS with the control sequence (19,22). However, K289M behaves differently in the presence of the APC sequence. The catalytic rate of K289M loses its dependence on the leaving group p*K*_a4_ as shown by the LFER slope of -0.07 (Supplementary Table S5, Figure 2B, blue). A LFER slope approaching zero indicates that the K289M rate-limiting TS is insensitive to leaving group basicity and that phosphodiester bond formation is not completely rate-limiting in the presence of the APC sequence (16). These results indicate that the activation energy of a pre-catalytic step becomes greater than that of phosphodiester bond formation in the presence of the APC sequence. Moreover, the difference in the LFER slopes indicates that the energetics of the TS catalyzed by K289M are sequence context-dependent.

### The two nucleotides on each side of the templating base are sufficient for the K289M transition state sequence specificity

Next, we examined whether the nucleotides that are adjacent to the templating base influence the sequence context-specific catalysis by K289M. We utilized two DNA substrates, control in APC and APC in control, in which the two nucleotides on each side of the templating base of the one sequence are altered to those found in the other sequence (Table 1). Consistent with the previous results, the catalytic rate of WT pol β is dependent on the leaving group basicity (Supplementary Table S4). Most importantly, the chemical TS catalyzed by WT exhibits little sequence context-dependence (LFER slopes of −0.52 for control in APC and −0.42 for APC in control, Figure 2A). On the other hand, the LFER slopes for K289M indicate that the nucleotides flanking the templating base impact the sequence context-specificity exhibited by K289M (Supplementary Table S5). For example, the LFER slope for the APC in control sequence is −0.06 and is similar to that of the APC sequence (−0.07); the LFER slope for the control in APC sequence is −0.24 and is similar to that of the control sequence (−0.27) (Figure 2B). This indicates that the nucleotides immediately adjacent to the templating base influence the activation energy of the reaction intermediates of K289M. The AACAA sequence context likely contributes to an increase in the activation energy of a pre-catalytic step to make it at least partially rate-limiting for K289M pol β. Therefore, the combination of the K289M pol β variant with a specific DNA sequence provokes an alteration of one or more pre-catalytic reaction steps, which likely results in reduced dNTP substrate selectivity, given that K289M is a mutator polymerase with the AACAA DNA sequence (34).

### Pre-catalytic conformational changes are slow for K289M with the control sequence

The polymerase domain of DNA pol β is organized into three subdomains, the thumb, palm, and fingers subdomains, resembling a right-handed structure (Figure 1) (41,42). The thumb subdomain is responsible for binding DNA, the palm subdomain contains the active site residues, and the fingers subdomain interacts with the incoming dNTP. Crystal structures of DNA pol β have revealed that the fingers subdomain undergoes a large conformational change to prepare the enzyme for the nucleophilic attack upon binding of the correct nucleotide (42,43). As a residue of helix N within the fingers subdomain, Lys289 moves during fingers closure once the dNTP is bound to the enzyme. In order to monitor fingers closing, we labelled the fingers subdomain with the fluorophore IAEDANS at residue 303 and also the DNA with Dabcyl, such that when the fingers close, the fluorescence from the AEDANS is quenched as it approaches the DNA (Supplementary Figure S2). Using this system, we showed that the fingers subdomain of DNA pol β undergoes at least two pre-catalytic conformational changes, fingers closing and a NCS, the identity of which is unknown (15,27). These conformational changes are thought to optimize active site geometry upon dNTP binding (Scheme 1) and have been suggested to influence selectivity in Klenow fragment (44), DinB (45), Dpo4 DNA polymerases (46) and DNA pol β (15).

**Figure 3.**
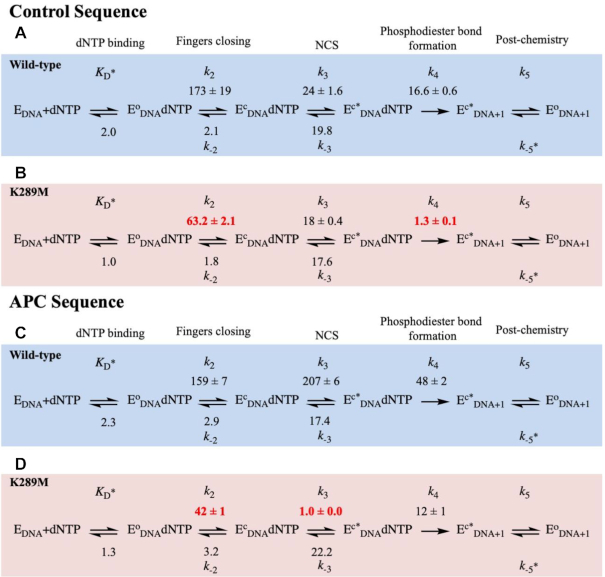
Modelled rates of conformational changes. FRET traces were modelled using KinTek Global Explorer to get rates for WT (blue) and K289M (red) in the presence of the Control (**A** and **B**) and APC (**C** and **D**) sequences. In this reaction mechanism, dNTP binds to the binary complex (E_DNA_) to form an open ternary complex (E^o^_DNA_ dNTP). The fingers subdomain undergoes two sequential conformational changes (fingers closing (E^c^_DNA_ dNTP) and NCS (E^c*^_DNA_ dNTP)) before product formation (E^c*^_DNA+1_). Finally, the enzyme undergoes a post-chemical conformational change to form E^o^_DNA+1_. Standard error of the fit is reported. Rate constants are in units of s^−1^ except for *K*_D_ (μM), *k*_-5_ (μM^−1^ s^−1^).

Here, we employ our FRET system to monitor the rates of pre-catalytic conformational changes of WT and K289M pol β during catalysis (Supplementary Figure S2) using the Control and APC DNA sequences (Table 1). Additionally, we measure the reverse rates of fingers closing and the NCS (Scheme 1) by performing a competition assay between a labelled ternary complex (containing labelled pol β, DNA substrate and dGTP) and a dark binary complex (containing pol β and DNA substrate) (Supplementary Table S5 and Supplementary Figure S3) as described in the Methods section (15). The results were modeled using KinTek Global Explorer in accordance to Scheme 1. Furthermore, the models were evaluated by Fitspace contour analysis as described in Methods (Figure 3, Supplementary Figure S4).

In the presence of the control sequence (Figure 3A, blue), WT pol β undergoes a rapid forward fingers closing (*k*_+2_, 173 ± 19 s^−1)^ with a slow reverse rate (*k*_-2_, 2.1 ± 0.0 s^−1^). Subsequently, the NCS occurs at a rate (*k*_+3_, 24 ± 2 s^−1^) approaching the rate of phosphodiester bond formation with a slow reverse rate (*k*_−3_, 19.8 ± 0.5 s^−1^). The rate of phosphodiester bond formation remains slower than the pre-catalytic conformational changes (*k*_+4_, 16.6 ± 0.6 s^−1^). The modelled rate of phosphodiester bond formation is similar to the rate measured biochemically, which brings confidence to the model (Supplementary Table S1, *k*_pol_ of 12.6 ± 1.1 s^−1^).

Lys289 is located in the fingers subdomain of DNA pol β (Figure 1A), thus, a mutation at this position may influence conformational changes during the catalytic cycle. The rate of fingers closing of K289M with the control sequence is 63 ± 2 s^−1^, (Figure 3B, red), which is ∼3-fold slower than the rate of the WT with the control sequence (173 ± 19 s^−1^) (Figure 3A). The reverse rates of fingers closing are slow and similar for WT and K289M pol β (Figure 3A and B).

Moreover, in the presence of the control sequence, the rate of the forward NCS (*k*_+3_, 18 ± 0.4 s^−1^) is similar to the reverse rate of the NCS (*k*_−3_, 17.6 ± 0.3 s^−1^) for K289M (Figure 3B). The rates of fingers closing and the NCS are faster than the rate of phosphodiester bond formation (*k*_+4_, 1.3 ± 0.1 s^−1^) for K289M (Figure 3B). Phosphodiester bond formation for K289M (*k*_*+*4_; 1.3 ± 0.1 s^−1^) is ∼13-fold slower than that of WT (*k*_*+*4_; 16.6 ± 0.6 s^−1^) with the control sequence (Figure 3A and B). The modelled rate of phosphodiester bond formation is similar to the rate obtained biochemically (Supplementary Table S1, *k*_pol_ 1.5 ± 0.1 s^−1^), bringing confidence to the models.

### The rates of pre-catalytic conformational changes are altered with the APC sequence

In the presence of the APC sequence (Figure 3C), the WT exhibits a rapid rate for fingers closing (159 ± 7 s^−1^) similar to the control sequence (173 ± 19 s^−1^) (Figure 3A). However, the NCS occurs at a faster rate for the APC sequence (*k*_+3_, 207 ± 6 s^−1^) (Figure 3C) as compared to the control sequence (24 ± 6 s^−1^) (Figure 3A). This indicates that the sequence context influences the rate of this step for WT pol β. Importantly, phosphodiester bond formation is slower than the pre-catalytic conformational changes for WT pol β in the presence of the APC sequence.

In the presence of the APC sequence (Figure 3D), the rate of fingers closing for K289M is 42 ± 1.1 s^−1^ (Figure 3D), which is similar to the rate of fingers closing with the control sequence (63 .2 s^−1^) (Figure 3B). However, fingers closing for K289M is ∼4-fold slower than that of WT (Figure 3C) with the APC sequence, which is similar to the differences observed between WT and K289M for the rates of fingers closure with the control sequence. In the presence of APC sequence, the NCS for K289M is 1.0 ± 0.0 s^−1^ (Figure 3D), which is 18-fold slower than with the control sequence. Remarkably, the rate of the NCS for K289M with the APC sequence (Figure 3D) is ∼200-fold slower than that of the WT (207 ± 6 s^−1^) with this sequence (Figure 3C). Additionally, the forward rate of the NCS of K289M (*k*_+3_, 1.0 ± 0.0 s^−1^) is slower than the reverse NCS (*k*_−3_, 22.2 ± 0.5 s^−1^), which indicates that the intermediate formed, E^c^*_DNA_dNTP, is unstable and that its formation is unfavourable. Most importantly, the rate of the NCS is slower than the rate of phosphodiester bond formation (*k*_+4_, 12 ± 1 s^−1^) (Figure 3D).

**Figure 4.**
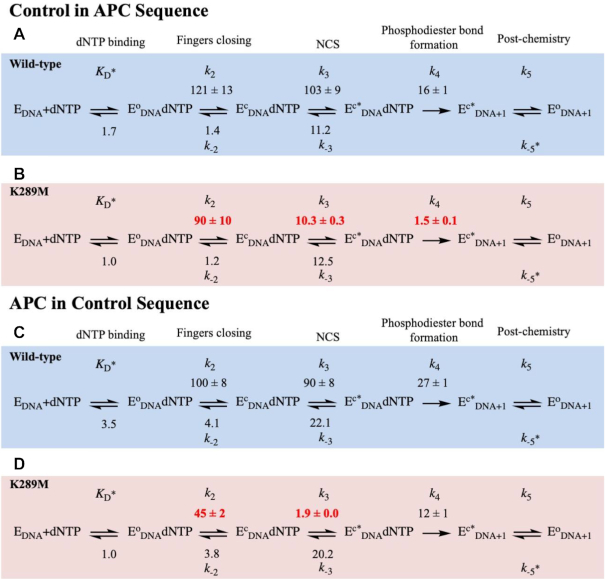
Modelled rates of conformational changes for Control in APC and APC in Control sequences. WT (blue) and K289M (red) were modeled using KinTek Global Explorer in the presence of the Control in APC (**A** and **B**) and APC in Control (**C** and **D**), sequences. Standard error of the fit is reported. Rate constants are in units of s^−1^ except for *K*_D_ (μM), *k*_−5_ (μM^−1^ s^−1^).

We also characterized the pre-catalytic conformational changes for WT pol β with the control in APC and APC in control DNA substrates. The rates of fingers closing are similar for WT with all four sequences (Figures 3A, 3C, S5S and S5C). The rate of the NCS for WT is fastest with the APC sequence (207 s^−1^; Figure 3B), followed by control in APC (103 s^−1^, Figure 4A) and APC in control sequences (90 s^−1^; Figure 4C) and is slowest for the control sequence (24 s^−1^; Figure 3A). In contrast, for K289M, the rate of the NCS is fastest with the control sequence (18 s^−1^; Figure 3B), followed by control in APC (10.3 s^−1^; Figure 4B). The NCS is very slow for K289M in the presence of the APC sequence (1.0 s^−1^; Figure 3D) and the APC in control sequence (1.9 s^−1^; Figure 4D). Importantly, the rate of the NCS for K289M in the presence of the APC in control sequence is slower than that of phosphodiester bond formation (Figure 4D), similar to what was observed for K289M with the APC sequence (Figure 3D).

### The sequence context and the templating base are both important factors in determining the energetics of the K289M catalytic intermediates

**Figure 5.**
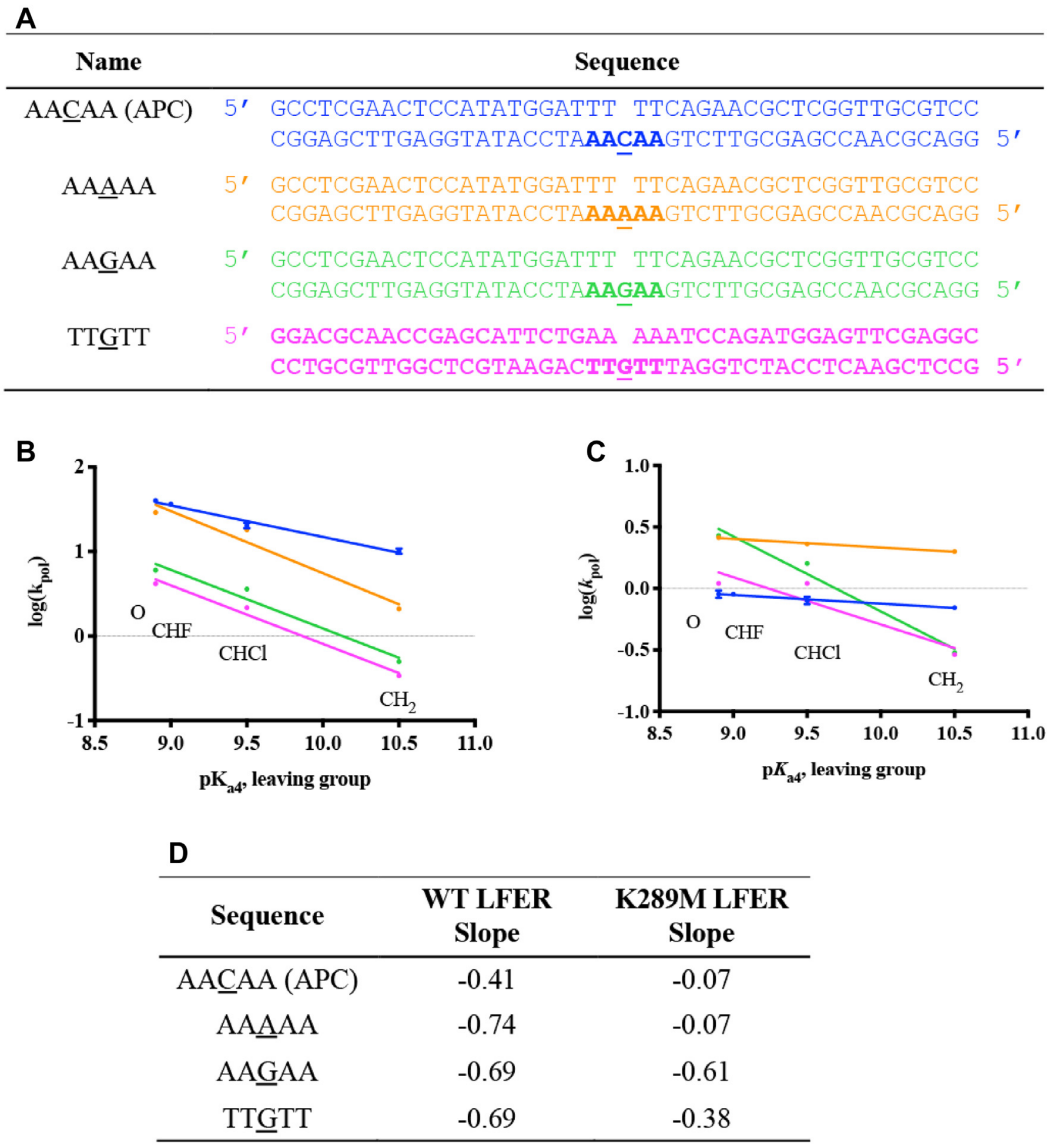
Sequence context-specificity of the linear free energy relationship. (**A**) Variations of the APC sequence were used where the templating base was changed to either A or G; also, the sequence complementary to the APC sequence was used containing TTGTT instead of AACAA. The linear free energy relationships between log(*k*_pol_) and p*K*_a4_ for WT (**B**) and K289M (**C**) are shown and the slopes for each of the sequences are listed (**D**).

In order to understand the contribution of the surrounding and templating bases to the sequence context-specificity of K289M, we varied the APC sequence to produce the sequences listed in Figure 5A. A sequence complementary to the APC sequence, containing TTGTT on the templating strand near the gap (Figure 5A), was used to understand the effect of the purine repeats in the primer terminus on the sequence context-specificity of K289M. Both the WT and K289M had a negative LFER slope in the presence of this sequence (−0.69 and −0.38, respectively) (Figure 5). This confirms that the WT exhibits a rate-limiting phosphodiester bond formation step regardless of the sequence. Moreover, the negative slope for K289M indicates that the sequence of the primer terminus affects the energetics of the catalytic intermediates of the cancer variant. As a result, the phosphodiester bond formation becomes rate-limiting for K289M in the presence of the TTGTT sequence.

We also tested the effect of the templating base on the sequence context-specificity of K289M by varying the templating base of the APC sequence from C into either A or G. In the presence of a templating G (AAGAA) and incoming dCTP, the WT and K289M have negative LFER slopes of −0.69 and −0.61, respectively, indicating that phosphodiester bond formation is rate-limiting. However, when the templating base is an A within a run of A (AAAAA) along with an incoming dTTP, slippage is observed for both WT and K289M (Supplementary Figure S5). As a result, multiple product bands were formed. We estimated the rates of polymerization by summing all product bands. Extracting the rate of a single nucleotide incorporation was not possible given the unknown equilibrium between substrates and products in the reaction due to primer-template slippage. Plotting of these estimated rates demonstrates that the rate of polymerization decreases with increasing basicity of the incoming dNTP analogue only for WT (LFER slope = −0.74) and not K289M (LFER slope = −0.07). Unfortunately, these results cannot be modeled with Kintek Global Explorer. In summary, these results indicate that both the APC sequence context and the templating base are factors that affect the energetics of the reaction intermediates of K289M and may contribute to its fidelity.

### Fidelity is sequence context-dependent

Fidelity is used as a measure of misincorporation frequency by DNA pol β. In our study, fidelity is obtained from the catalytic specificity of dGTP incorporation (*k*_pol_/*K*_D(dNTP)_) relative to the catalytic specificity of misincorporation of dCTP opposite templating C and in the context of the control and APC sequences, which is presented in Table 2. The fidelity of WT is 12 × 10^4^ in the presence of the control sequence, however, there is a 5-fold reduction in the presence of the APC sequence. The fidelity of WT in the presence of the other two sequences (control in APC and APC in control) is similar to what we observe for the control sequence.

In the presence of the control sequence, K289M has a fidelity similar to that of WT pol β. In contrast, there is a ∼31-fold decrease in fidelity of K289M in the presence of the APC sequence compared to the fidelity of WT pol β with the control sequence.

Lastly, fidelity of the WT and K289M pol β in the presence of the varied APC sequences was measured using the efficiency of correct incorporation as compared to misincorporation of dGTP (Table 2). In the presence of the templating G sequence, both the WT and K289M maintain fidelity relative the control sequence. However, in the presence of the AAAAA sequence, there is a 5-fold reduction in fidelity relative to the control sequence for both the WT and K289M.

## DISCUSSION

Work by us and others (13–18,34) show that the inherent properties of pol β and other polymerases in combination with the sequence context of the DNA substrate are critical for dNTP selection by pol β. Here, we reveal that the mechanism of sequence context-dependent fidelity features a NCS that precedes phosphodiester bond formation. We show that the K289M mutation alters the enzyme such that it is unable to accommodate the APC DNA substrate in a manner that promotes accurate DNA synthesis.

### The transition state catalyzed by K289M is sequence context-dependent

Using a dNTP toolkit that monitors the dependence of the TS on phosphodiester bond formation, we show that WT pol β catalyzes the formation of a rate-limiting chemical TS in the presence of either the control or APC sequence contexts. In contrast, a mutation from Lys289 to Met results in the formation of a rate-limiting chemical TS in a sequence context-dependent manner. Specifically, the rate of phosphodiester bond formation is slower than pre-catalytic conformational changes in K289M in the presence of the control sequence but not in the presence of the APC sequence. Upon further analysis of the kinetics of the K289M variant, we show that a pre-catalytic NCS is slower than phosphodiester bond formation in the presence of the APC sequence but not with the control sequence. Interestingly, the rate of the NCS increases dramatically for WT in the presence of the APC versus control sequence (207 versus 24 s^−1^, respectively) and fidelity is decreased by about 5-fold. Since both WT and K289M pol β have reduced fidelity in the presence of the APC sequence, we propose that the pre-catalytic conformational changes of these enzymes are a determining factor of fidelity. Our results indicate that the rate of NCS needs to be within a specific range in order for the enzyme to correctly select a nucleaotide and that conformational changes are impacted by the sequence context of the DNA substrate.

### Lys289 is necessary for rapid fingers closing and phosphodiester bond formation and for accommodating various DNA sequence contexts

**Figure 6.**
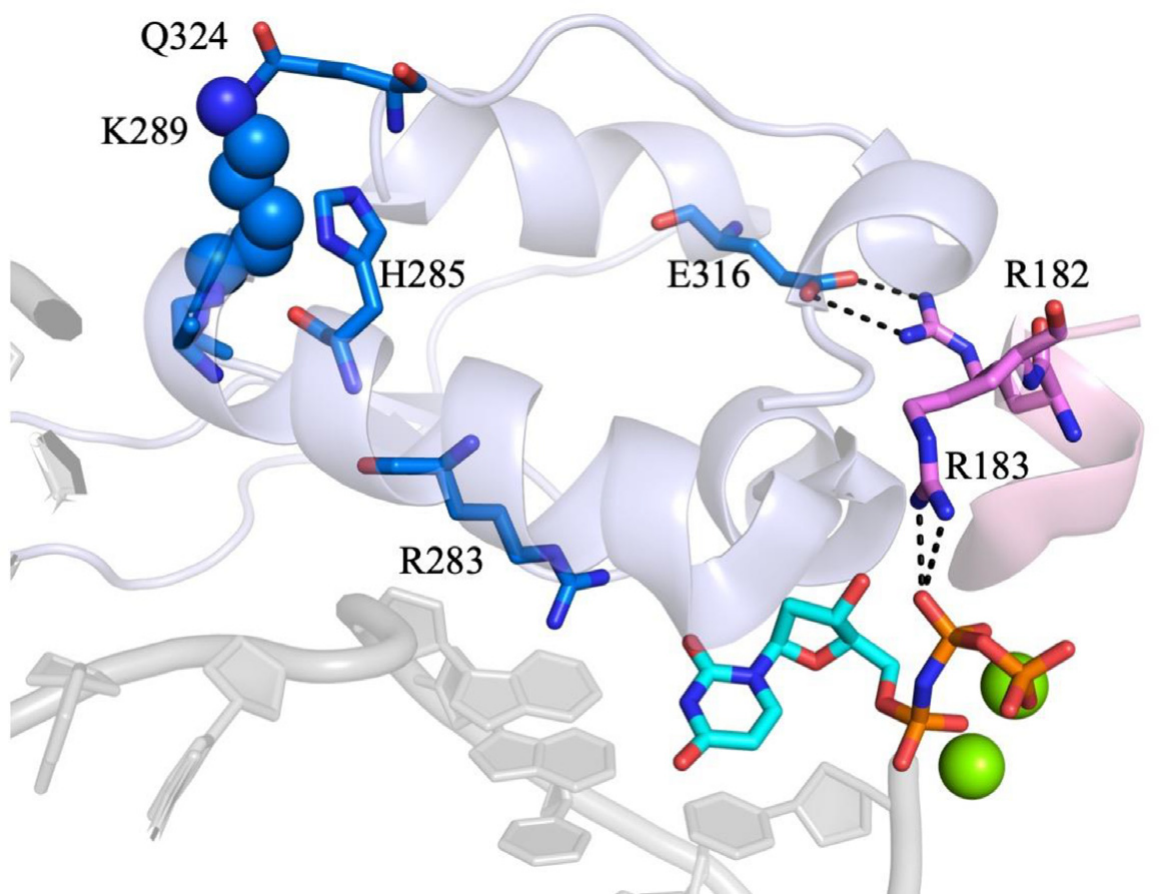
The indirect interactions of K289 with the active site. K289 is closely located to Q324 of the fingers subdomain, which is located in the same helix as E316. This residue interacts with R182, which is adjacent to R183 and forms electrostatic interactions with the β, γ-bridging group of the incoming dNTP. Additionally, K289 is part of helix N undergoes a large conformational change upon binding of the correct dNTP. Helix N contains residues that have been implicated in the fidelity of pol β. Most importantly, R283 interacts with the templating base of the DNA along with the incoming dNTP (PDB code 4KLE).

Fingers closing has been shown to occur via the movement and rotation of helix N of the fingers subdomain to enclose the DNA and active site in order to prepare the enzyme for a nucleophilic attack (42,47). Lys289 is located at the distal end of helix N of the fingers subdomain (Figure 6). Mutating the basic residue, Lys289, into a hydrophobic Met may destabilize helix N, which can influence both the rate of fingers closing and, consequently, the rate of phosphodiester bond formation. Because helix N contains residues that have been shown to interact with the DNA backbone, including N279, R283, M284 and H285, we propose that these interactions are destabilized by the K289M mutation. Moreover, many of the residues in helix N have been shown to be important for activity and fidelity, given their interactions with DNA and the templating base (Figure 6). As a result of the K289M mutation, the active site may not be able to properly accommodate the APC and perhaps other contexts, leading to reduced fidelity (Table 2). Notably, when the rate of the NCS is significantly altered, fidelity is reduced. Therefore, we propose that the interactions of residues of helix N are critical for the execution of the NCS under circumstances where fidelity is maintained.

### The non-covalent step is dependent on the proper alignment of the primer terminus at the active site

Previous work from our laboratory and others (27,44,48) demonstrates the presence of the NCS, which occurs after fingers closing and prior to phosphodiester bond formation. Specifically, having A/T repeats adjacent to the gap is shown to significantly affect the NCS. We propose that this sequence-context dependence results from differential base stacking and base pairing within the DNA and the incoming dNTP. Watson-Crick base pairing and base stacking have long been suggested to govern substrate selection by DNA polymerases (for a review see (49)). Earlier work from the Goodman lab shows that base stacking plays an absolutely critical role during selection of the dNTP substrate by DNA polymerases (50,51). Notably, the primer terminus of the APC sequence consists of three pyrimidines (TTT). Pyrimidines are known to stack in DNA more weakly than purines (52) and we suggest that this difficulty in stacking in combination with weak base pairing between T and the complementary A at the primer terminus may contribute to the instability of 3′OH position, which may result in changes in the NCS rate. In support of this, LFERs in the presence of the sequence complementary to the APC sequence (containing TTGTT, Figure 5) exhibit slopes of −0.69 and −0.38 for WT and K289M, which are similar to slopes of the control sequence (Figure 5B and C). These results indicate that the composition of the primer terminus influences the energetics of the K289M reaction intermediates where the thymidine repeat of the APC sequence reduces the rate of a pre-catalytic step while the adenine repeats in the primer terminus of the TTGTT sequence have a rate-limiting phosphodiester bond formation step. Moreover, we tested the effect of the templating base in the context of the APC sequence on the LFER slope for WT and K289M. We varied the templating base of the APC sequence (AACAA) into either A or G (Figure 5). The results shown in Figure 5 indicate that the WT has a steep negative slope independent of the sequence context and of the templating base. However, K289M lacks a slope in the presence of the APC sequence (AACAA) and is estimated to lack a slope with the AAAAA sequence (Slope of −0.07 for both sequences) whereas it exhibits a negative slope with the AAGAA sequence. The AAAAA sequence is particularly important because this nucleotide repeat is present at 30 sites within the APC gene including sites of mutation hotspots (35). Moreover, previous work on microsatellite instability in the APC gene indicates that the error frequency for pol β is 50-fold higher in the presence of a sequence of adenine (A_8_) (53). Though it is important to understand the effect of this sequence on the fidelity of pol β, our conclusions are limited by estimated rates resulting from primer-template slippage.

Our results indicate that in addition to the sequence context of the primer DNA, the templating base is also important in defining the energy levels of the TS. This DNA effect in combination with an inherent defect in fingers closing for K289M pol β leads the NCS to become slower than phosphodiester bond formation. Moreover, it has been previously reported that for Klenow fragment, the occupancy at the active site is governed by conformational changes in the DNA in the templating region, which can be regulated by the incoming dNTP (54), therefore, this is used as a check for fidelity. A change in the template conformation was observed in the presence of the correct but not the incorrect nucleotide. Interestingly, the ‘real-time’ crystal structures of pol β harboring mismatched bases within the active show that during the first observable change in structure, the primer terminus exhibits a localized highly dynamic state and poor electron density (28). In combination, these results indicate that a key component of substrate discrimination is positioning of the primer terminus. Therefore, it is possible that the inability of K289M to undergo rapid fingers closing and NCS would permit greater DNA flexibility, specifically at the primer terminus, which would not support efficient catalysis and lead to reduced frequency of correct incorporation.

### Fidelity is governed by pre-catalytic conformational changes

Several *in vitro* studies with pol β and other DNA polymerases show that these enzymes exhibit DNA sequence context-dependent preferences for the induction of mutations (14,55–60). More recent studies from our laboratory have provided evidence that mutator variants of pol β display error-prone DNA synthesis activity within different DNA sequence contexts (13,18,34,61,62). The work described here suggests that dynamic pre-catalytic conformational changes of pol β, and perhaps other DNA polymerases, occur in response to the nature of the DNA primer template and the incoming dNTP in order to promote error-free DNA synthesis.

## Supplementary Material

gkz1076_Supplemental_File
