# Supplementary material for: A pre-catalytic non-covalent step governs DNA polymerase β fidelity
Source: Nucleic Acids Res. 2019 Nov 16;47(22):11839–49. doi: 10.1093/nar/gkz1076 (PMC7145665; doi:10.1093/nar/gkz1076)
Supplement: gkz1076_Supplemental_File [file gkz1076_supplemental_file.docx]

**A Pre-Catalytic Non-Covalent Step Governs DNA Polymerase β Fidelity**

Khadijeh S. Alnajjar ^1^, Ivan S. Krylov^2^, Amirsoheil Negahbani^2^, Boris A. Kashemirov^2^, Ji Huang^1^, Mariam Mahmoud^1^, Charles E. McKenna^2^, Myron F. Goodman^2,3^ and Joann B. Sweasy^1,4^*

^1^ Department of Cellular and Molecular Medicine, University of Arizona, Tucson, Arizona 85724, United States

^2^ Department of Chemistry, University of Southern California, Los Angeles, California 90089, United States

^3^ Department of Biological Sciences, University of Southern California, Los Angeles, California 90089, United States

^4^ University of Arizona Cancer Center, Tucson, Arizona 85724, United States

* To whom correspondence should be addressed. Tel: +520 626 5549; Fax: +520 626 6898; Email: jsweasy@email.arizona.edu

**Supporting Material**

| **Table S1. Sequence-context dependent kinetics of WT and K289M with the parent dGTP.** | | | | | | | | | | | |
| --- | --- | --- | --- | --- | --- | --- | --- | --- | --- | --- | --- |
|  |  | M-N *^a^* | *k*_pol_ (s^-1^) *^b^* | | | D *k*_pol_ (c/i) *^c^* | *K*_D_ (µM) | | | D *K*_D_ (i/c) *^d^* | |
| WT | Control | C-G | 12.6 | ± | 1.1 | 194 | 2.0 | ± | 0.1 | |  |
|  |  | C-C | 0.07 | ± | 0.01 |  | 1277 | ± | 202 | | 639 |
|  | APC | C-G | 35.5 | ± | 1.2 | 161 | 2.3 | ± | 0.3 | |  |
|  |  | C-C | 0.22 | ± | 0.01 |  | 340 | ± | 68 | | 148 |
|  | Control in APC | C-G | 22.1 | ± | 1.0 | 737 | 1.7 | ± | 0.3 | |  |
|  |  | C-C | 0.03 | ± | 0.002 |  | 456 | ± | 108 | | 268 |
|  | APC in Control | C-G | 28.5 | ± | 5.2 | 750 | 3.5 | ± | 0.8 | |  |
|  |  | C-C | 0.04 | ± | 0.003 |  | 389 | ± | 123 | | 111 |
| K289M | Control | C-G | 1.52 | ± | 0.08 | 76 | 1.0 | ± | 0.2 | |  |
|  |  | C-C | 0.02 | ± | 0.002 |  | 778 | ± | 182 | | 778 |
|  | APC | C-G | 0.90 | ± | 0.1 | 18 | 1.3 | ± | 0.3 | |  |
|  |  | C-C | 0.05 | ± | 0.005 |  | 267 | ± | 88 | | 205 |
|  | Control in APC | C-G | 1.40 | ± | 0.09 | 74 | 1.0 | ± | 0.3 | |  |
|  |  | C-C | 0.02 | ± | 0.002 |  | 758 | ± | 267 | | 758 |
|  | APC in Control | C-G | 1.50 | ± | 0.10 | 30 | 1.5 | ± | 0.8 | |  |
|  |  | C-C | 0.05 | ± | 0.001 |  | 766 | ± | 89 | | 511 |
| *^a^* M-N is the templating base (C) and incoming parent dNTP. dGTP is the correct and dCTP is the incorrect nucleotide. | | | | | | | | | | | |
| *^b^* Standard deviation is reported for three or more repeats.  *^c^* Discrimination at *k*_pol_: *k*_pol(correct)_/*k*_pol(incorrect)_. | | | | | | | | | | | |
| *^d^* Discrimination at *K*_D_: *K*_D(incorrect)_/*K*_D(correct)_. | | | | | | | | | | | |

| **Table S2. Rates of the reverse conformational changes.** | | | | | | | | |
| --- | --- | --- | --- | --- | --- | --- | --- | --- |
| Sequence | Enzyme | *k*_-2_*^a^*  E^c^_DNA_dNTP 🡪 E^o^_DNA_dNTP | | | *k*_-3_  E^c*^_DNA_dNTP 🡪 E^c^_DNA_dNTP | | | |
| Control | WT | 2.1 | ± | 0.0 | 19.8 | ± | 0.5 | |
|  | K289M | 1.8 | ± | 0.0 | 17.6 | ± | 0.3 | |
| APC | WT | 2.9 | ± | 0.1 | 17.4 | ± | 0.3 | |
|  | K289M | 3.2 | ± | 0.1 | 22.2 | ± | 0.5 | |
| Control in APC | WT | 1.4 | ± | 0.0 | 11.2 | ± | 0.2 | |
|  | K289M | 1.2 | ± | 0.0 | 12.5 | ± | 0.2 | |
| APC in Control | WT | 4.1 | ± | 0.1 | 22.1 | ± | 0.4 | |
|  | K289M | 2.8 | ± | 0.1 | 20.2 | ± | 0.4 | |
| *^a^* GraphPad Prism was used to obtain the reported rates along with the standard error. *k*_-2_ and *k*_-3_ are the reverse steps of fingers closing and NCS, respectively | | | | | | | | |
|  | | | | | | | |  |

| **Table S3. Binding affinity of WT and K289M to DNA.** | | | | | | | |
| --- | --- | --- | --- | --- | --- | --- | --- |
| Sequence | WT *^a^* | | | | K289M | | |
| Control | 2.0 | ± | 0.2 | 3.8 | | ± | 0.7 |
| APC | 2.6 | ± | 0.1 | 4.2 | | ± | 0.4 |
| Control in APC | 2.0 | ± | 0.1 | 3.3 | | ± | 0.6 |
| APC in Control | 3.0 | ± | 0.1 | 5.2 | | ± | 0.8 |
| *^a^* In units of nM as measured by the electrophoretic mobility shift assay (± standard deviation). | | | | | | | |

**Table S4. Single turnover kinetics of WT.**

| Sequence | Analogue | p*K*_a4_ | *k*_pol_ (s^-1^) *^a^* | | | *K*_D_ (µM) | | | | Specificity *^b^* (*x*10^6^ M^-1^s^-1^) | |  |
| --- | --- | --- | --- | --- | --- | --- | --- | --- | --- | --- | --- | --- |
| Control | O | 8.9 | 12.6 | ± | 1.1 | | 2.0 | ± | 0.1 | | 6.30 | |
|  | CHF | 9.0 | 10.2 | ± | 1.1 | | 4.2 | ± | 1.0 | | 2.40 | |
|  | CHCl | 9.5 | 5.90 | ± | 0.1 | | 0.5 | ± | 0.4 | | 11.8 | |
|  | CH_2_ | 10.5 | 2.20 | ± | 0.4 | | 0.5 | ± | 0.1 | | 4.30 | |
| APC | O | 8.9 | 35.5 | ± | 1.2 | | 2.3 | ± | 0.3 | | 15.4 | |
|  | CHF | 9.0 | 36.4 | ± | 2.0 | | 7.5 | ± | 0.7 | | 4.90 | |
|  | CHCl | 9.5 | 20.3 | ± | 1.8 | | 7.8 | ± | 1.6 | | 2.60 | |
|  | CH_2_ | 10.5 | 9.00 | ± | 0.7 | | 7.1 | ± | 1.2 | | 1.30 | |
| Control in APC | O | 8.9 | 22.1 | ± | 1.0 | | 1.7 | ± | 0.3 | | 13.0 | |
|  | CHF | 9.0 | 19.9 | ± | 1.2 | | 2.2 | ± | 0.6 | | 9.00 | |
|  | CHCl | 9.5 | 11.9 | ± | 0.7 | | 5.6 | ± | 1.4 | | 2.10 | |
|  | CH_2_ | 10.5 | 3.10 | ± | 0.2 | | 2.2 | ± | 0.1 | | 1.40 | |
| APC in Control | O | 8.9 | 28.5 | ± | 1.5 | | 3.5 | ± | 0.8 | | 8.10 | |
|  | CHF | 9.0 | 25.1 | ± | 2.2 | | 5.2 | ± | 1.5 | | 4.80 | |
|  | CHCl | 9.5 | 21.7 | ± | 0.8 | | 7.5 | ± | 1.1 | | 2.90 | |
|  | CH_2_ | 10.5 | 6.30 | ± | 0.8 | | 6.9 | ± | 2.0 | | 0.90 | |
| *^a^* Values are reported as mean ± standard deviation of three or more repeats. *^b^* Specificity is *k*_pol_/*K*_D_. | | | | | | | | | | | | |

**Table S5. Single turnover kinetics of K289M.**

| Sequence | Analogue | p*K*_a4_ | *k*_pol_ (s^-1^) *^a^* | | | *K*_D_ (µM) | | | Specificity *^b^* (*x*10^6^ M^-1^s^-1^) |
| --- | --- | --- | --- | --- | --- | --- | --- | --- | --- |
| Control | O | 8.9 | 1.52 | ± | 0.08 | 0.99 | ± | 0.20 | 1.54 |
|  | CHF | 9.0 | 1.50 | ± | 0.10 | 1.30 | ± | 0.40 | 1.15 |
|  | CHCl | 9.5 | 1.18 | ± | 0.07 | 2.85 | ± | 0.80 | 0.41 |
|  | CH_2_ | 10.5 | 0.58 | ± | 0.06 | 2.20 | ± | 0.80 | 0.26 |
| APC | O | 8.9 | 0.90 | ± | 0.10 | 1.30 | ± | 0.30 | 0.69 |
|  | CHF | 9.0 | 0.90 | ± | 0.01 | 1.60 | ± | 0.20 | 0.56 |
|  | CHCl | 9.5 | 0.80 | ± | 0.10 | 2.00 | ± | 0.40 | 0.40 |
|  | CH_2_ | 10.5 | 0.70 | ± | 0.01 | 1.70 | ± | 0.10 | 0.41 |
| Control in APC | O | 8.9 | 1.42 | ± | 0.09 | 1.04 | ± | 0.30 | 1.37 |
|  | CHF | 9.0 | 1.50 | ± | 0.10 | 1.20 | ± | 0.50 | 1.25 |
|  | CHCl | 9.5 | 1.01 | ± | 0.09 | 6.50 | ± | 2.30 | 0.16 |
|  | CH_2_ | 10.5 | 0.61 | ± | 0.06 | 1.60 | ± | 0.60 | 0.38 |
| APC in Control | O | 8.9 | 1.53 | ± | 0.13 | 1.53 | ± | 0.80 | 1.00 |
|  | CHF | 9.0 | 1.36 | ± | 0.04 | 1.04 | ± | 0.30 | 1.31 |
|  | CHCl | 9.5 | 1.37 | ± | 0.06 | 10.0 | ± | 2.10 | 0.14 |
|  | CH_2_ | 10.5 | 1.17 | ± | 0.05 | 4.75 | ± | 0.87 | 0.25 |
| *^a^* Values are reported as mean ± standard deviation of three or more repeats. *^b^* Specificity is *k*_pol_/*K*_D_. | | | | | | | | | |


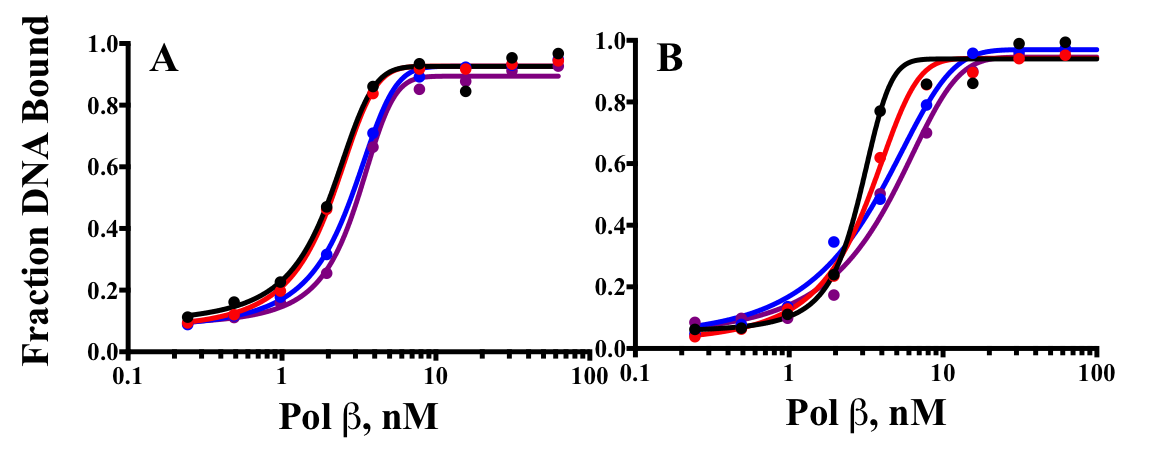


**Figure S1**. **DNA binding affinity**. Binding affinity of WT (A) and K289M (B) to Control (Black), APC (Blue), Control in APC (Red), and APC in Control (Purple) sequences by the electromobility shift assay. The binding affinity to DNA is independent of the sequence for WT and K289M pol β.


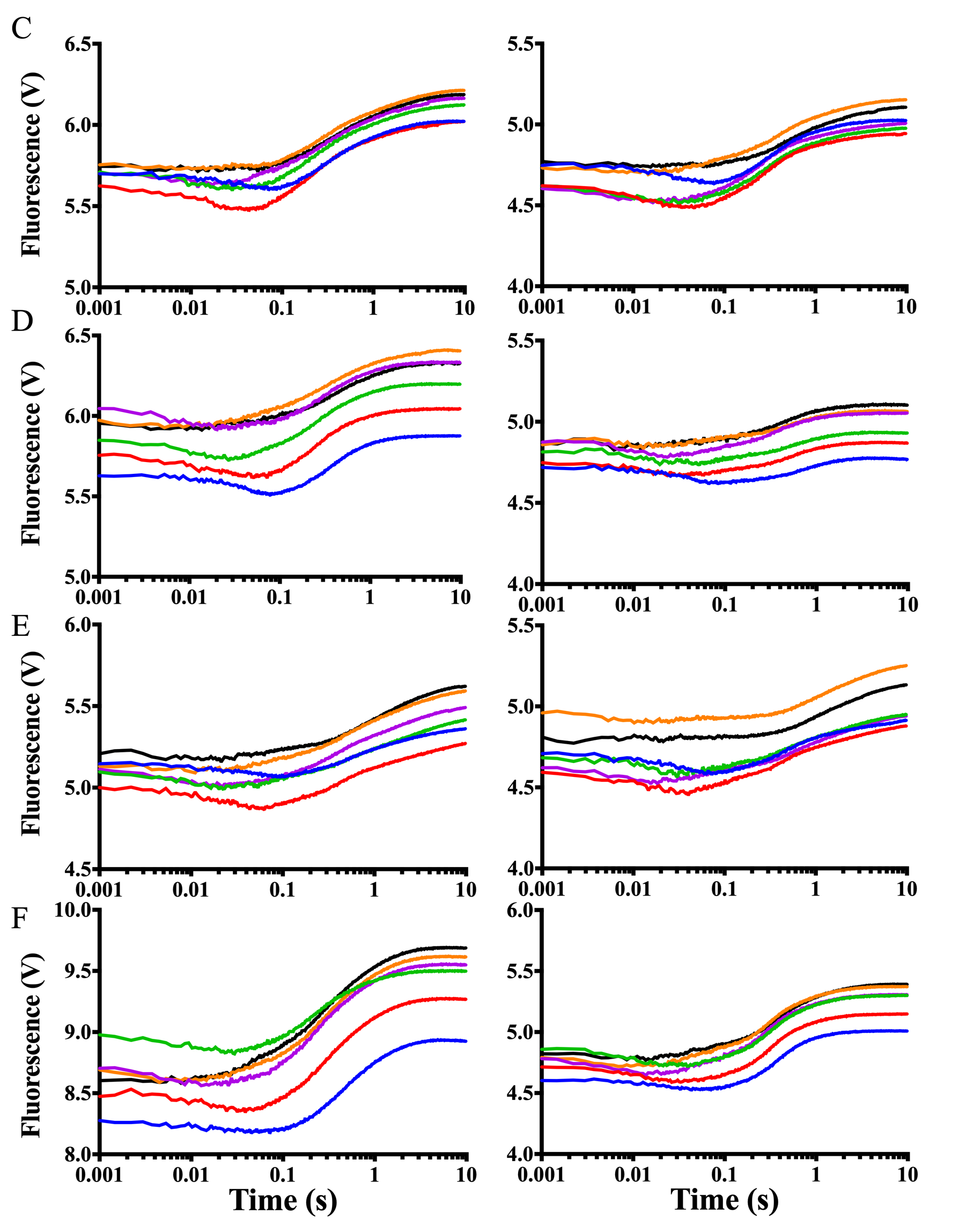

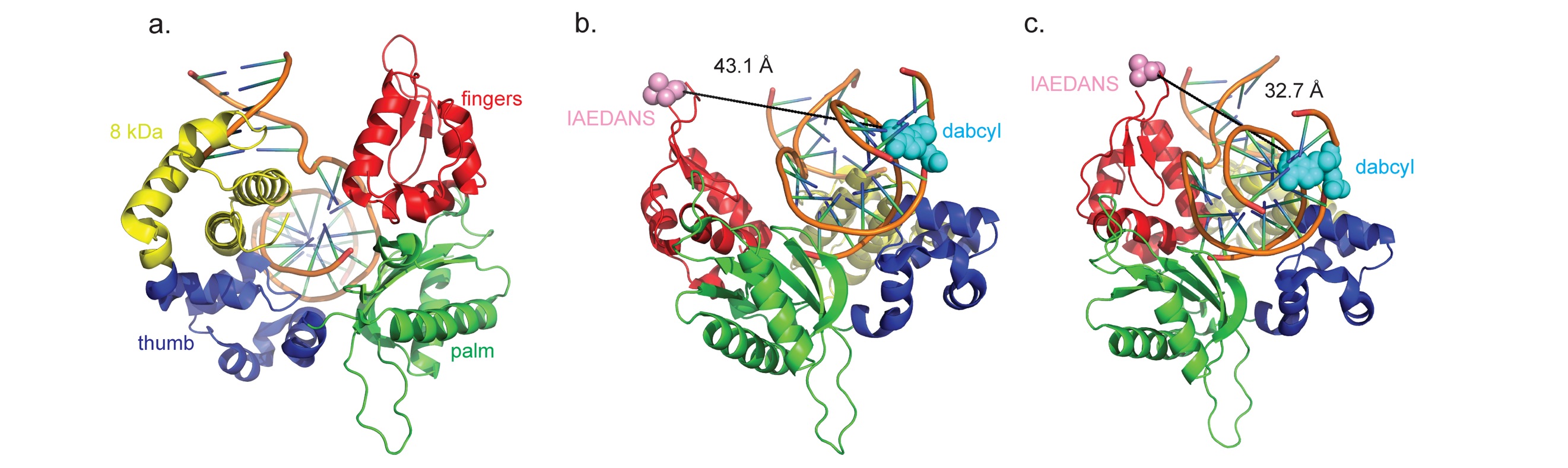


A

B

**Figure S2. Sequence context-specificity of conformational changes as measured by FRET.**

The fingers subdomain was labeled with AEDANS at position V303C and DNA was labeled with Dabcyl at position T-8 relative to the gap. The two dyes are separated by 43.1 Å in the open binary conformation (A) and 32.7 Å in the closed ternary complex (B) (PDB code 3ISB and 4KLE). Changes in fluorescence emission were recorded as a function of time upon rapidly mixing the WT (Left) or K289M (Right) in the presence of various sequences with dGTP using stopped-flow fluorometer. The sequences used are: Control (C), APC (D), Control in APC (E), and APC in Control (F) and the dGTP concentrations used are: 1 µM in blue, 2.5 µM in red, 5 µM in green, 10 µM in purple, 25 µM in orange, and 50 µM in black. Each trace represents an average of at least 10 shots.


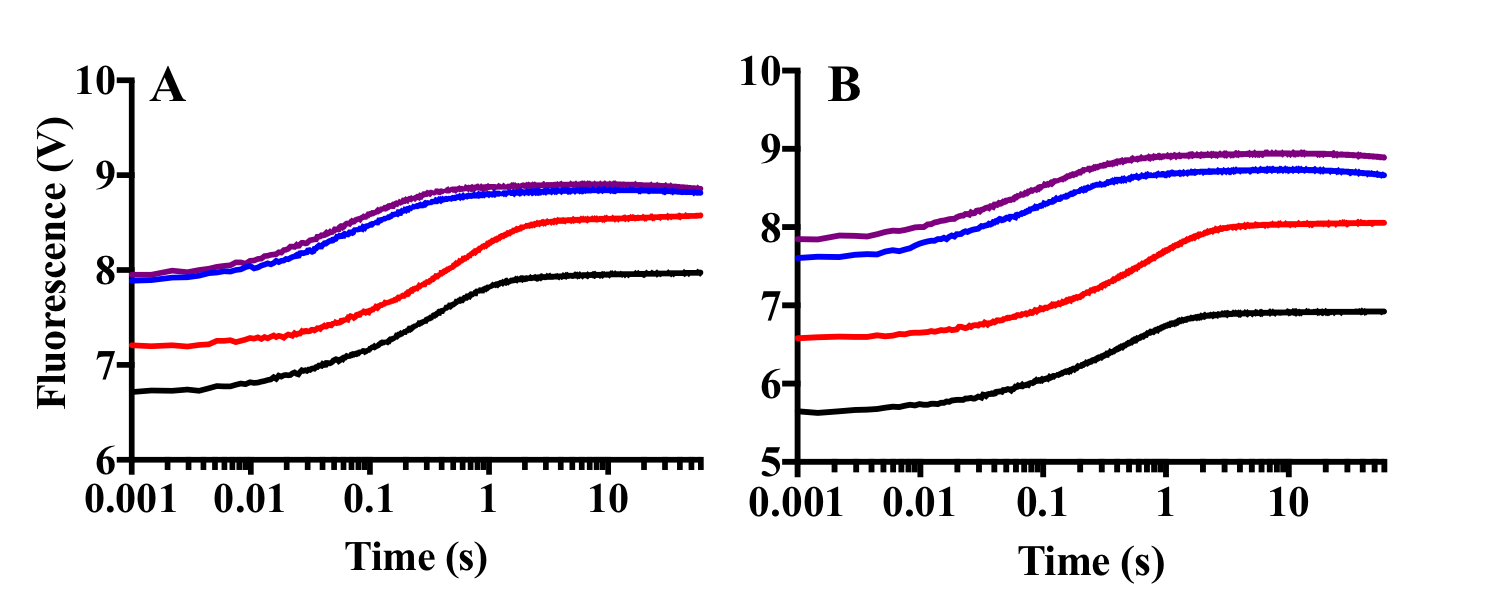


**Figure S3. Competition assay for re-opening the closed ternary complex.** Reverse rates were obtained for steps 2 and 3 of the reaction mechanism for WT (A) and K289M (B) in the presence of Control (Black), APC (Blue), Control in APC (Red), and APC in Control sequences (Purple) using Stopped-flow fluorometer. Traces were fit to a two exponential fit using GraphPad Prism and rates obtained were fixed in KinTek Global Explorer to bring confidence in the fits. Each trace represents an average of at least 20 shots.

**Figure S4. 2D FitSpace contour analysis**. Confidence contour analysis of the fits with all ten pairwise combinations of the five unknown rates (*k*_+2_, *k*_+3_, *k*_+4_, *k*_+5_, *k*_-5_) in the presence of each of the sequences for WT and K289M, respectively: Control (A and B), APC (C and D), Control in APC (E and F), and APC in Control (G and H). The values for each pair of parameters in each panel were varied while the others were held constant and the sum square error (SSE) was evaluated. This analysis provides a range of values for each parameter that achieves a good fit. The heat map represents SSE_x,y_/SSE_min_, where 1 is the best fit and lowest SSE.


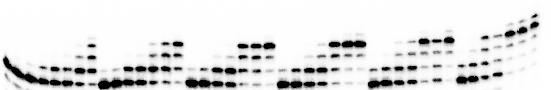

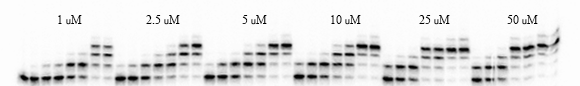


**A**

**B**

Time (s) 0 0.02-10 0.02-10 0.02-10 0.02-10 0.02-10 0.02-10

Time (s) 0 0.05-30 0.05-30 0.05-30 0.05-30 0.05-30 0.05-30

**Figure S5.** **Polymerization reaction in the presence of the A repeat sequence**. With increasing reaction time between pol β, the gapped DNA substrate, and with increasing dTTP concentration, multiple product bands are formed (up to 4 product bands, substrate labeled with arrow). The concentration of the parent dTTP used is labeled on top and the reaction times used are 0.02-10 sec for WT (A) and 0.05-30 sec for K289M (B). The formation of multiple product bands impaired our ability to accurately describe the dependence of phosphodiester bond formation on the p*K*_a4_ of leaving group.
